# Human gene expression sensitivity according to large scale meta-analysis

**DOI:** 10.1186/1471-2105-10-S1-S56

**Published:** 2009-01-30

**Authors:** Pei Hao, Siyuan Zheng, Jie Ping, Kang Tu, Christian Gieger, Rui Wang-Sattler, Yang Zhong, Yixue Li

**Affiliations:** 1 Bioinformatics Center, Key Lab of Systems Biology, Shanghai Institutes for Biological Sciences, Chinese Academy of Sciences, Shanghai 200031, PR China; 2 Shanghai Center for Bioinformation Technology, 100 Qinzhou Road, Shanghai 200235, PR China; 3 School of Life Sciences, Fudan University, Shanghai 200433, PR China; 4 College of life science and biotechnology, Shanghai Jiaotong University, Shanghai 200240, PR China; 5 Institute of Epidemiology, Helmholtz Zentrum München, German Research Center for Environmental Health, 85764 Neuherberg, Germany

## Abstract

**Background:**

Genes show different sensitivities in expression corresponding to various biological conditions. Systematical study of this concept is required because of its important implications in microarray analysis etc. J.H. Ohn et al. first studied this gene property with yeast transcriptional profiling data.

**Results:**

Here we propose a calculation framework for gene expression sensitivity analysis. We also compared the functions, centralities and transcriptional regulations of the sensitive and robust genes. We found that the robust genes tended to be involved in essential cellular processes. Oppositely, the sensitive genes perform their functions diversely. Moreover while genes from both groups show similar geometric centrality by coupling them onto integrated protein networks, the robust genes have higher vertex degree and betweenness than that of the sensitive genes. An interesting fact was also found that, not alike the sensitive genes, the robust genes shared less transcription factors as their regulators.

**Conclusion:**

Our study reveals different propensities of gene expression to external perturbations, demonstrates different roles of sensitive genes and robust genes in the cell and proposes the necessity of combining the gene expression sensitivity in the microarray analysis.

## Background

Genes show divergent expression patterns under various biological conditions, therefore a common task for biologists and biostatisticians is to find the differentially expressed genes between different conditions, such as treatment versus control, or normal versus abnormal, so as to identify the condition specific gene markers [1-3]. With the high throughput microarray technology, expression levels of several thousands of genes can be detected simultaneously and compared in parallel between numerous biological samples [4,5], thus facilitating the study of gene expression-environment interactions.

Although external environment has important influences on the gene expression profiles, genes show different susceptivity. An intuitive example is the housekeeping genes which are required for the maintenance of the basal cellular functions [6] and believed to constitutively express in most of the tissues, though different expression levels can be observed (data not shown). This hypothesis was once used to identify HK genes, and promoted the understanding of HK genes [7-9].

Recently J.H. Ohn et al [10] constructed a non-directed bipartite perturbation network to study the yeast gene expression sensitivity to external perturbations. Through an 'excess retention' approach [11], they show significant differences between perturbation sensitive genes and perturbation resistant genes in protein interaction network, regulatory network and functional categories. As an exploratory work their study was based on the transcriptional profiling of gene deletion experiments of yeast and got very significant results. It is worthy of generalizing such kind of idea to human genes based on general biological condition variations to obtain a global view of the intrinsic properties of human gene expression as a response to perturbations. For this purpose we selected human gene expression data resulted from divergent experiments stored at the GEO database [12] and developed a meta-analysis method to study gene expression sensitivity globally. Regarding to our calculations it was found that the human genes show different expression sensitivities and can be categorized into sensitive or robust groups, according to the properties of how they response to the perturbations. Furthermore, in order to know the detail properties about related functions and interaction properties of both gene groups we assigned them onto protein-protein interaction networks and gene transcriptional regulatory networks. It was discovered that the robust genes tend to be involved in essential cellular processes. In contrast, the sensitive genes perform their functions diversely. We also found even if genes from both groups show similar geometric centrality by coupling them onto integrated protein networks, the robust genes have higher vertex degree and betweenness than that of the sensitive genes. Finally, an interesting fact has been found, not alike the sensitive genes, the robust genes share less transcription factors as their regulators. These facts discovered here maybe are useful for deciphering functions and related regulatory mechanisms of genes.

## Methods

### Data collection and preprocessing

All the GDS data sets of Affymetrix HGU133a platform in the GEO database [12] were downloaded to incorporate as many as biological samples. The reason why we chose the Affymetrix HGU133a platform is that, it is one of the most widely used platforms, i.e. there are far more data sets of HGU133a (236 data sets) than that of HGU133plus2.0 (69 data sets) in GEO. Data sets with less than 10 arrays were discarded. For each sample, the expression values that were below 10 were truncated to 10, and then were logarithmic transformed (base 2). The expression values of all probes for a given gene were reduced to a single value by taking the maximum expression value in each sample.

### Calculate the matrix of standard deviations

For every data set, calculate the standard deviation (sd) for each gene g. Because the data sets are heterogeneous, expression standard deviations from different data sets for gene g can not be compared directly, therefore the sd of every data set were rank ordered, generating a rank sd matrix.

### Statistical analysis

If gene g is sensitive to the environment or biological conditions, relative big standard deviation is expected for its expression levels and oppositely for the robust genes. Moreover, if this trend can be observed in multiple data sets, it's more confident. Based on this hypothesis, we test for every gene g in the sd rank matrix if the sd rank concentrates at the bottom or top of the whole gene list, corresponding to expression sensitiveness and robustness respectively. Specifically, suppose there are N genes on the array and M data sets, the sd rank of gene g is a vector of length *M *S = (sdr_1_, sdr_2_,...., sdr_M_), and the sd rank order of all the genes is a list L = (1, 2..., N), we test the relative positions of S in L. For every S, set the initial Sensitive Score (SS) to 0, and then walk down the list L, if a sd rank in S is encountered at position i, SS is incremented by *P*_*hit*_, otherwise SS is decremented by *P*_*miss*_. The *P*_*hit *_and *P*_*miss *_is given as,

$$\begin{array}{cc} P_{hit\left(i\right)}=\frac{\left|sdr_{i}-N/2\right|}{\sum_{sdr\in S}\left|sdr-N/2\right|} & P_{miss}=\frac{1}{N-M} \end{array}$$

The final SS is the maximum deviation from zero. SS ranges from -1 to 1, and more closer to 1, more expression robust and vice versa. To evaluate the significance of an observed SS, a null distribution of SS is generated by randomly permutating the L. By 1000 random permutations of L, SSnull was computed for each SS and the nominal P value was assigned as the negative or positive portion of the SSnull corresponding to the observed sign of SS.

Denote the SS from random permutations as SSπ, the observed SS distribution as SSα. For a specific SS > 0, calculate the percentage of SSπ > SS which SSπ > 0; calculate the percentage of SSα > SS which SSα > 0, the FDR (False Discovery Rate) for SS is computed as ratio of the two percentages when SS > 0, similarly if SS < 0.

This algorithm resembles the GSEA algorithm [13,14] and the Sensitivity Score corresponds to a Kolmogorov-Smirnov like statistic.

## Results

Though the Affymetrix HGU133a microarray does not represent all the human genes, by calculating the Sensitivity Score (SS) we can identify gene classes which are assumed to be rich in expression sensitive and robust genes. We investigated the genomic characteristics of the respective groups, including functional enrichment, centralities in the protein interaction network and regulations in the transcriptional regulatory network.

### Assignment of expression robust and sensitive genes

**Figure 1:**
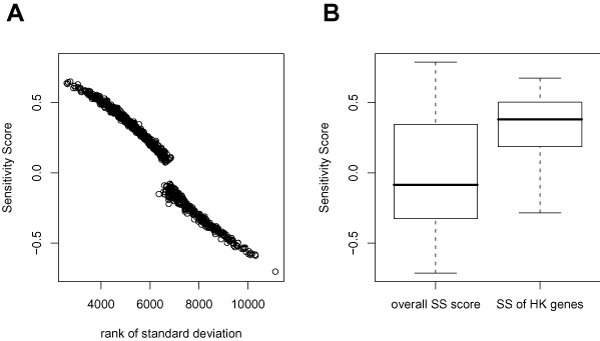
**Validation of SS as a sensitivity measure**. The figure A demonstrates strong correlations between the SS and the average rank order of gene expression standard deviations in the studied data sets. The figure B shows the housekeeping genes (HK genes) have significant higher SS, indicating their higher expression robustness.

We first validated if the SS could reflect the relative gene expression variations. We calculated the average rank order of gene expression standard deviations in the studied data sets and found a strong negative correlation with SS (γ = -0.97, p < 2.2e-16) (Figure 1A).

Housekeeping genes (HK genes) have constitutive expressions [6], therefore comparative small expression variations of HK genes are expected under the numerous biological conditions. Eisenberg et al. identified 575 human HK genes [8] with a transcriptional profiling data set [15]. We compared the SS of aforementioned HK genes with the overall SS and found that the HK genes have significant higher SS (Wilcox rank sum test, p < 2.2e-16) (Figure 1B).

Based on the above observations that the SS is a reasonable measurement for gene expression sensitivity, we selected two groups of genes as representative expression robust (661 genes) and sensitive genes (441 genes) based on the SS score cutoff 0.55, -0.5 respectively (see supplementary text for the discussion of statistical significance of SS). The functional analysis results are not sensitive to the exact SS cutoff. To evaluate the robustness of this categorization to different microarray platforms, we conducted a similar analysis on the HGU133plus2.0 microarray data sets following the same pipeline and test if the robust/sensitive genes remain robust/sensitive. Although the HGU133plus2.0 microarray represents much more genes, the result shows that the robust/sensitive genes identified from HGU133a microarrays are still robust/sensitive on the HGU133plus2.0 microarray (Additional File 1).

### Functional annotations of expression robust and sensitive genes

**Figure 2:**
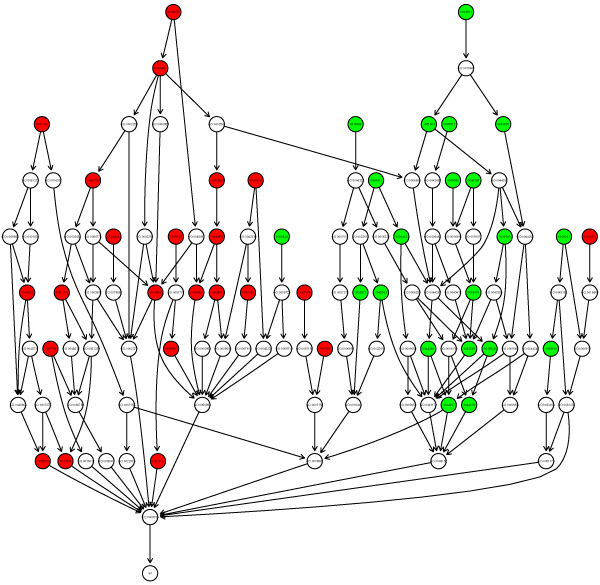
**Enriched Gene Ontology distribution of the two gene groups (biological process)**. The enriched GO terms are colored red for sensitive genes and green for robust genes. These two groups have distinct enriched GO distributions, indicating their different functions in the cell.

Gene ontology [16] annotations of robust and sensitive genes are useful to reveal respective roles of these genes in the cell. We conducted enrichment analysis in "biological process" and "cellular compartment" for the two gene classes. From the resultant induced GO graph, robust genes and sensitive genes have obvious distinct function distributions, as shown in the Figure 2.

Specifically most of the enriched biological processes of the sensitive genes refer to cell responses to environmental perturbations, e.g. immune responses, cell-cell signalling, while those of the robust genes refer to some cell essential activities, e.g. protein, RNA metabolic process, translation (Table 1). Correspondingly, the enriched cellular compartments of the sensitive genes are extracellular region, while the robust genes are preferentially located in ribosome, nucleus etc.

**Table 1:** Enriched biological processes. The table shows some of the enriched biological processes of the sensitive and robust genes respectively.

Robust Genes		Sensitive Genes	
Enriched Biological Process	P value	Enriched Biological Process	P value

protein metabolic process	1.75e-07	immune response	1.64e-26

translation	1.27e-06	inflammatory response	3.32e-14

RNA metabolic process	1.39e-06	cell-cell signaling	1.02e-07

### Comparisons of robust and sensitive genes in protein interaction network and transcriptional regulatory network

Topological characteristics of protein interaction network are associated with many gene properties, e.g. gene essentiality[17], gene duplicability [18]. Here we focus on the centrality of robust and sensitive genes in the network. To make reliable inferences from the comparison result, we used a high-quality protein interaction data [19,20]. We also confirmed the result with the HPRD [21] interaction data (Additional file 1).

**Figure 3:**
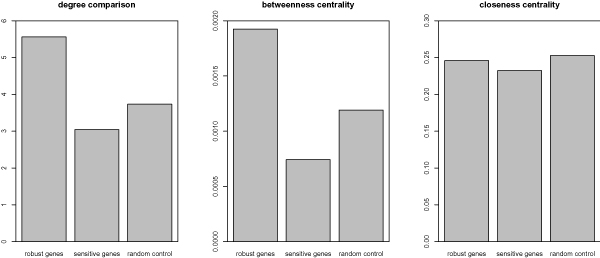
**Centrality comparisons of robust and sensitive genes**. Three centrality measures were calculated and compared. A group of genes were randomly sampled and compared to the sensitive and robust genes. As the figure shows, though the robust genes present no significant higher closeness centrality, they have higher degree centrality (p < 0.05) and betweenness centrality (p < 0.05) than the sensitive genes.

Three widely used centrality measures were calculated, degree, betweenness and closeness. Besides, we randomly sampled a group of genes from the protein interaction network as the control group. As the result shows (Figure 3), among the three groups, the robust genes have the highest degree and betweenness, while the sensitive genes have the lowest. Interestingly, similar closeness was observed for all the groups.

Jeong et al. have shown that protein lethality is correlated with its degree in the protein interaction network [22]. This correlation implicates the bigger importance of robust genes to the cell system, consistent with the higher betweenness which is originally designed to measure the influence of a node over the spread of information through the network [23]. Closeness measures gene's geometric centrality in the network. The comparison of closeness indicates that no group is organizationally more central than others. It is noteworthy that similar results were obtained when comparing the geometric centrality of essential genes and non-essential genes in yeast with a measure called 'excentricity' [24]. This phenomenon is believed to be due to the function compensations [24].

Transcriptional regulatory network differs with the protein interaction network that they reflect different layers of cellular activities. Transcription factors, which bind to the gene upstream promoter regions, have significant influences on the gene expressions. Therefore, a natural question is, do the robust genes and the sensitive genes have different extent of regulation by transcription factors? To answer this question, we compared the upstream binding transcription factors of these two gene classes. We used the TRANSFAC database to build the regulatory network. Though it is far from complete, it is the most reliable and confident data source till now. For the 641 robust genes, there are totally 26 transcription factors recorded in the TRANSFAC database that can bind to promoter regions of them, while for the 441 sensitive genes, the number of regulatory transcription factors rises to 155. This result is consistent with the previous report [10] that the expression of sensitive genes is under more regulations.

## Discussion

Gene expression sensitivity measures gene's responses to the external environment on the transcriptome level. In this study, we proposed a large scale meta-analysis strategy to categorize expression robust and sensitive genes. Further we found these two gene classes show significant differences in various aspects, including functions based on Gene Ontology classification [16], centralities in protein networks and regulations by transcription factors.

The Gene Ontology analysis shows distinct functional differences between the robust and sensitive genes. The enriched biological processes of robust genes concentrate on the cellular essential processes, for instances, protein, mRNA metabolic process, translation, ubiquitin cycle etc, while for sensitive genes, the enriched biological processes concentrate on some cell "response" processes to the surrounding environment, like the immune responses, cell-cell signalling. Such functional preferences confirm the implications of these gene classes and reflect their different roles in the cell. Centrality analysis reveals that although they have similar geometric positions in the interactome, they show different local characterization (degree) and different weight for the spread of information (betweenness) in the protein network. Jeong et al. have reported the correlation between protein lethality and its degree in the protein interaction network [22], and another study shows the high-betweenness proteins are more likely to be essential [25]. Together with the function analysis, we come to the conclusion that there are connections between gene expression sensitivity and the genes' impact on the system. More transcription factors were found to bind to sensitive genes. This result is analogous to the finding that yeast non-essential genes are regulated by more transcription factors compared with essential genes [17]. It seems the essential process related genes tend to have simpler regulatory mode, which makes the cell more stable.

Though our study incorporated large volume of microarray data, there are several potential limitations. For example, more data was generated for the hot spots of the biological research, thus decreased the diversity of the experimental samples in our study. In addition, the affymetrix HGU133a microarray represents 13441 genes on the chip, however, the number of human genes is estimated to be between 20,000 and 25,000 [26]. Another restriction to our observations is, the current map of protein interactions and gene regulations is far from complete.

## Conclusion

A major challenge of microarray analysis is interpreting the biological relevance of changes in expression [27]. However, the current approaches tend to select genes with the largest changes in expression. Our analysis suggests that genes have different propensities corresponding to perturbations and such propensities should be considered in the gene expression data analysis.

Understanding gene expression sensitivity has important implications for choosing biomarkers, drug targets etc from transcriptional profiling data. Though we explored the general characteristics of expression robust and sensitive genes, the underlying mechanisms of gene transcription sensitivity still represent further challenges.

## Competing interests

The authors declare that they have no competing interests.

## Authors' contributions

PH, SZ and CG devised the algorithm. SZ implemented the molecular network analysis and wrote the paper in collaboration with PH. PJ and RW validated the analysis pipeline with HGU133plus2.0 microarrays. KT collected the expression data. YZ and YL conceived and directed this work. All authors read and approved the final manuscript.

## Supplementary Material

Additional file 1 A Microsoft word file including the discussion of the significance of the sensitivity score and the confirmation of the analysis results. Click here for file
