# Supplementary material for: Human gene expression sensitivity according to large scale meta-analysis
Source: BMC Bioinformatics. 2009 Jan 30;10(Suppl 1):S56. doi: 10.1186/1471-2105-10-S1-S56 (PMC2648786; doi:10.1186/1471-2105-10-S1-S56)
Supplement: Additional file 1 — A Microsoft word file including the discussion of the significance of the sensitivity score and the confirmation of the analysis results. [file 1471-2105-10-S1-S56-S1.doc]

**Supplementary Text**

**1. Discussions about the statistical significance of Sensitivity Scores.**

Nominal P value and FDR were calculated following the protocol described in the main text. The FDR distribution is shown in the next figure.

It is a skew distribution, and according to a usual cutoff, most of the genes show significant Sensitivity Score (9795 genes with FDR < 0.05). However, we think this result is reasonable. The null hypothesis is that the standard deviations of gene expression are randomly distributed in the list L, the FDR indicates it is not the case for most of the genes. Standard Kolmogorov-Smirnov test was performed for validation. As shown in the below picture, after a Benjamini & Yekutieli FDR adjust, the standard Kolmogorov-Smirnov test generates similar FDR distribution.

As described in the main text, we sorted the SS and selected two groups with the top or bottom SS as representative expression robust and sensitive genes. Though the SS cutoff seems arbitrary (SS > 0.55 or SS < -0.5), similar results were obtained according to different cutoffs (data not shown).

**2. Validation from HGU133plus2.0 microarray data**

We downloaded all the HGU133plus2.0 GDS data sets from the GEO database and assigned sensitivity score for every gene on the array, following the same analysis pipeline described in the main text. Because the HGU133plus2.0 array represents much more genes than HGU133a array (20253 vs 13442), we aim to check if the robust/sensitive genes identified from the latter platform remain robust/sensitive as well on the former platform.

The above left figure illustrates the SS rank of robust genes on the HGU133plus2.0 arrays, and similarly the right figure illustrates the SS rank of sensitive genes on the HGU 133plus2.0 arrays. Bigger rank indicates bigger SS. It is clearly shown that the robust genes have comparative big SS on the HGU133plus2.0 array and similarly for the sensitive genes, indicating the consistency between these different microarray types.

**3. Topological properties of robust and sensitive genes**

We focus on the centrality of robust and sensitive genes in the protein interaction network. Three centrality measures were calculated: degree, betweenness centrality and closeness centrality.

1. Degree

Degree is defined as the number of direct neighbors of a node v in the network G.

1. Betweenness centrality

Betweenness centrality is calculated as the fraction of number of shortest paths that pass through each node, and then normalized by b=b/(n-1)(n-2) where n is the order of network.

1. Closeness centrality

Closeness centrality is calculated as (1/average distance to all nodes) for one node v in the network. From its definition, closeness centrality reflects the geometric position of a node in the graph.

The control group order for high quality PPI network is 200, and for HPRD network the order is 500. The control genes were randomly sampled from the network and the centrality measurements were calculated. This process was repeated for 100 times.

Here we show the comparisons of the three centrality measures calculated from HPRD PPI network.

Statistical test shows the robust genes have significantly higher degrees and betweenness centrality than the sensitive genes. No significant differences were observed for the three groups.
